# A qualitative study of Ghanaian pediatric oncology nurses’ care practice challenges

**DOI:** 10.1186/s12912-021-00538-x

**Published:** 2021-01-12

**Authors:** Ruth Nimota Nukpezah, Fatemeh Khoshnavay Fomani, Marzieh Hasanpour, Alireza Nikbakht Nasrabadi

**Affiliations:** 1 Department of Pediatric Nursing, School of Nursing and Midwifery, International Campus-Tehran University of Medical Sciences, Tehran, Iran; 2 Department of Pediatric Nursing, School of Nursing and Midwifery, Tehran University of Medical Sciences, Tehran, Iran; 3 Department of Pediatric Nursing, NIDCAP Professional, Spiritual Health Branch of Research Center of the Quran; Hadith and Medicine, School of Nursing and Midwifery, Tehran University of Medical Sciences, Tehran, Iran; 4 Department of Medical and Surgical Nursing, School of Nursing and Midwifery, Tehran University of Medical Sciences, Tehran, Iran

**Keywords:** Cancer, Children, Ghana, Challenges, Oncology nurses, Content analysis, Qualitative study

## Abstract

**Background:**

Pediatric cancer is a global problem, and some studies have emphasized that nurses caring for these children experience work-related challenges. This has caused many children diagnosed with cancer to have a prolonged hospital stay and suffer unnecessary pain. However, there is insufficient documentary evidence on this issue. This study aims to explore and understand the challenges faced by pediatric oncology nurses in caring for children in Ghana.

**Methods:**

An exploratory qualitative research design study was conducted from August 2019 to April 2020. The study was conducted at the pediatric oncology unit which is located at the Tamale Teaching Hospital (TTH), Ghana. The study was conducted among 14 Ghanaian pediatric oncology nurses who were purposively sampled. A semi-structured interview guide was used to collect data. The interviews were recorded, transcribed verbatim, and analyzed inductively using Elo and Kyngas content analysis approach. The criteria proposed by Guba and Lincoln were used to ensure the validity of the study.

**Results:**

From the analysis of participants transcripts, eight subcategories emerged from two major categories. The subcategories were; time-consuming care, low job motivations, inadequate logistics, work stress, reduced labour force, low knowledge level, lack of teamwork and the perception of contracting cancer.

**Conclusions:**

The results point to several organizational and personal constraints experienced by the nurses who work at the pediatric oncology ward. It is hoped that by addressing these challenges, it would lead to further improvement in the care that is provided to children with cancer. There is the need for the administrative managers of hospitals, government and other stakeholders to invest in human, material and financial resources for delivering childhood cancer care services.

**Supplementary Information:**

The online version contains supplementary material available at 10.1186/s12912-021-00538-x.

## What is already known about the topic?

The pediatric cancer care challenges experienced by nurses include; low technology of care and inadequate cancer education.

The Lack of managerial support, lack of nurses’ authority to change practice, Physical and psychological distress and lack of time have been frequently cited as challenges experienced by pediatric oncology nurses.

These challenges make children with cancer to be left with terminal diagnoses, unnecessary suffering and job dissatisfaction among nurses.

## What this study adds

The findings revealed that pediatric oncology nursing care practice is challenged with administrative and personal constraints.

The administrators of hospitals, governments and other stakeholders need to invest in human, material and financial resources to provide childhood cancer care services.

It is anticipated that by reducing the challenges identified in this current research, the survival rate and quality of life (QOL) of children with cancer will be improved, and nurses’ job satisfaction will be enhanced.

## Background

Cancer is one of the leading causes of death among children and adolescents worldwide, with approximately 300,000 children from birth to 19 years old being diagnosed with cancer each year [1]. Indeed, the nurse is viewed as a partner in the human care process with a high value placed on the relationship between the nurse and the patient [2]. Pediatric oncology nursing involves establishing intense interpersonal relationships, addressing the multiple and complex needs of children and caregivers, and being in constant contact with people’s suffering [3, 4]. Low and middle-income countries (LMICs) are cancer care resource-limited and this makes many children with cancer to be left with terminal diagnoses and unnecessary suffering [5]. The pediatric cancer care challenges in LMIC include low technology of care, inadequate cancer education [6, 7], and experiences of physical and psychological distress [8]. This claim is in line with the findings in a study conducted in Ghana, which opined that several factors within the work environment influence the performance of nursing care. The author stated that among all healthcare professionals in Ghana, nurses are the least satisfied with their remuneration, career development, management and work environment [9]. Renner and McGill (2016), also mentioned that in Ghana, the chances of survival for most patients diagnosed with cancer are usually bleak, being less than 20% [10]. This is due to challenges which were not well explained in the study result of Renner and McGill. Other studies acknowledged that the challenges involved in caring for patients are more intense in the pediatric oncology ward than other units in a hospital [11, 12]. Some studies have highlighted that in Egypt, nurses and pharmacists were exposed to hazardous drugs for cancer treatment, and in Iran, nurses showed changes in their mitochondrial parameters and there was cytotoxicity of their lymphocytes due to exposure to chemotherapy inhalation [5, 13]. These unfortunate incidents occurred because the hospital where they worked did not have adequate personal protective equipment’s for the nurses to work with. Similarly, limited specialized nursing training and inadequate staffing result in a longer hospital stay and more complications amongst patients in general [14, 15]. However, another study mentioned that the challenges of the pediatric oncology nurses are close to minimal [16].

Few researchers have examined this phenomenon in developing countries and just a few studies were found in industrialized nations [17–20]. Noteworthy, no study has been conducted to investigate into details, the daily challenges that the pediatric oncology nurses face in the oncology care units in Ghana. Thus, this study aimed to explore and understand the challenges that the pediatric oncology nurses experience when caring for children with cancer in Ghana. It is hoped that the results and recommendations from this study would help to better understand these complex challenges and inform solutions.

### Aim

To explore and understand the challenges that the pediatric oncology nurses experience when caring for children with cancer in Ghana.

## Methods

### Study design

We used an exploratory qualitative design. This method helps in bringing out the subjective realities and truths about the meaning and expressions of participants [21]. It further helps to better understand the challenges that the pediatric oncology nurses experience whiles caring for children with cancer in Ghana.

### Study setting

The research was conducted at the Tamale Teaching Hospital (TTH). This hospital is a Tertiary hospital and referral Centre for the five northern regions of Ghana. The Pediatric oncology unit was the Centre of the research and it is part and located in the Pediatric ward. The oncology unit has seven-bed capacity out of the forty-two total beds in the pediatric ward. All the interviews were conducted at the nurses’ private room.

### Characteristics of participants considered for inclusion

1) Nurses (male and female) who work at the Pediatric/Hematology Cancer Unit. 2) Participants who had at least two [2] years of work experience. 3) Participants who were willing to share their experiences 4) Participants who voluntarily agreed to participate in the research.

### Data collection

The purposive sampling technique was employed to select participants who met the inclusion criteria. Data was collected using a semi-structured interview guide as seen in the (the additional file) to obtain in-depth information. The guide was developed by the researchers based on the objective of the study. The interview guide file presents a list of questions that were developed by the research team according to the literature review and considerations of the current study aim. The guide consists of two categories which include the main and the probing qualitative interview questions. The questions asked were as follows: “Can you please tell me about your thoughts and feelings about your work of caring for children with cancer?”, “Tell me about the challenges you experience when taking care of children with cancer?” And probing questions such as “Could you please describe it more?”, was used so that the participants could share their opinion about their perceptions of their caregiving challenges and how these affected them in more details.

Data collection lasted from August 2019 to April 2020. Permission was obtained from the authorities of the Tamale Teaching Hospital. The ward in-charge at Pediatric/Hematology Cancer unit was first contacted. The information about the study aims and purpose was discussed. The ward in-charge assisted in recruiting the participants. All the potential participants identified were briefed about the purpose of the study and were invited to take part in the study. After which, oral and written consent was granted by the interviewees. The nurses’ sitting room was used because it was a serene location in the ward. The time chosen was convenient for the participants. The first author conducted the interviews in English and each interview lasted 45 to 60 min.

Digital tape recorder (Dictaphone) was used with the consent of the participants for the recordings of the interviews. Field notes were taken during and after the interviews. At the end of each interview session, all recordings were anonymized by the use of labels (P1 to P14).

After the interview, demographic data such as gender, age, education and number of years of experience were noted. Each interview conducted was immediately transcribed verbatim by the researcher. Data saturation (a point at which no new information emerges from the interviews) was reached by the time the 14th participant was interviewed.

### Data analysis

A content analysis method by Elo and Kyngas was used. This is a method that involves a systematic process of describing a specific phenomenon [22]. The three-stages of; preparation, organization and reporting of results, based on inductive qualitative analysis as described by Elo and Kyngas were followed [22]. The three-staged analysis process helped to obtain a detailed comprehension of the participant’s experiences.

The first *preparatory stage* deals with making sense of the data. Thus, the first author listened to the recorded participant’s data severally and transcribed it verbatim immediately after each interview. The transcribed text was reviewed several times to get a thorough understanding of the unit of analysis, that is “the challenges to pediatric oncology nurses care practices”.

In the second organization stage, the meaning units were clustered into codes by all the four authors (R.N.N.; A.N.N; F.K.F and M. H) to uncover similarities and discrepancies in the data. Coding means that quotes that explained all aspects of the study’s aim were written down, to get an overview of the participant’s experiences. Also, during the second stage, describing claims were formed from the quotes, after which the describing claim contents that were alike, were clustered together to form subcategories.

At the final, reporting stage, there was an abstraction of the findings. This was done by grouping subcategories with similar content to form categories. Thus, by collapsing content that was similar or dissimilar, sub-categories and categories were created as summarized in Table 2.

Throughout the process of analysis, subcategories and categories that differed were discussed until consensus was reached. We kept an audit trails of all the process, including all the changes made during the analysis process. We also ensured that all the processes specified by Elo and Kyngas were followed diligently [22].

### Ethical consideration

Ethical clearance was granted by the Research Ethics Board of School of Nursing and Midwifery, Tehran University of Medical Sciences, Tehran, Iran, on July 11, 2019 (approval code: IR. TUMS.VCR.REC.1398.273) and in Ghana by the Korlebu Teaching Hospital Research Ethics Board on December 31, 2019 (approval code: KBTH IRB/000127/2019). The participants were told about the purpose of the study and that participation was completely voluntary. They were also told that the findings from the study would be published in a reputable Journal and their anonymity and confidentiality were protected during the study. Participants were informed that they had the right to withdraw from the study at any time without penalties and that the interviews will be recorded. After all these thorough explanations, all the participants gave oral and written consent before participating in the study. Each interview was immediately transcribed and all the transcripts were identified with number codes and were kept in locked files in the investigator’s office. No ethical problem arose during the study.

### Methodological trustworthiness

The trustworthiness of a qualitative study is the extent to which the identified meanings accurately represent the participant’s perspectives [21]. The trustworthiness in the study was enhanced by enforcing the value of credibility, confirmability, dependability, and transferability [23]. Credibility means the degree of confidence that can be placed in the truth of the research findings [23]. The credibility of the study was ensured by long engagement with the participants, Investigator triangulation (using a four members research team approach) to mitigate the PI influence. Reflexivity was fostered through the use of research diary, by the use of the systematic analytical steps of Elo and Kyngas content analysis approach, and the use of in-depth Interview technique.

Confirmability refers to the degree of researcher’s neutrality in the interpretations [23]. This was achieved by the means of confirmability audit (audit trail of raw data, analysis notes, and Reflexive journaling) and using purposefully selected participants (information-rich cases) for in-depth study of their caregiving challenges.

Transferability shows how the qualitative researcher demonstrates that the research study’s findings are applicable to other similar contexts [situations, circumstances, populations, and phenomena] [23]. A thick description of the study settings and process involved in the study is provided, to enable transferability of the research findings to a similar context.

Dependability is the extent that the study could be repeated by other researchers and that the findings would be consistent [23].. To ensure the dependability of the study findings, the methodology steps (audit trail) used for data collection and analysis is adequately captured in the report, and also, we used the Consolidated criteria for reporting qualitative studies (COREQ), a 32-item checklist.

## Results

### Demographic characteristics of participants

Table 1, shows the demographic characteristics of participants. The study participants were 14 state registered nurses who work at the Pediatric/Hematology Cancer Unit at the Tamale Teaching Hospital, Ghana. Three [3] participants had a master’s degree in pediatric Nursing and the rest had B.Sc. degrees in Nursing. Three [3] participants had less than five years of work experience, six participants were ranged between 5 to 10 years in their work experience as pediatric oncology nurses and the rest of the participants had more than ten years work experience.

**Table 1:** Demographics characteristics of Participants

Variables	N (%)
**Gender**	
Male	9 (64.28)
Female	5 (35.71)
**Level of Education**	
BSc.	11 (78.57)
Masters	3 (21.42)
**Age**	
25–35 years	6 (42.85)
36–45 years	8 (57.14)
**Years of Experience as a nurse**	
Less than 5 years	3 (21.42)
5 to 10 years	6 (42.85)
More than 10 years	5 (35.71)

Authors Construct (2020)

Analysis of the interviews led to the identification of eight [8] identified challenges of the pediatric oncology nurses (Sub-categories), these challenges representing two [2] main category termed “Administrative constraints” and “Personal constraints”. An overview of the findings is shown in Table 2.

**Table 2:** Categories, Sub-Categories and Sample of Quotes

Categories	Sub- Categories	Sample of Quotes
Administrative constraints	➢ Time-consuming care. ➢ Lack of teamwork. ➢ Inadequate logistics. ➢ Work stress. ➢ Reduced labour force.	*“How do I manage my time? The problem is often not me; it relates to the manner of the oncology nursing care too. Many of us had experienced staying at the hospital even after our shift ended. I am often compelled to come to the hospital during my day-off. I feel it may be related to the time-demanding nature of oncology care. I feel that my colleagues in other hospital wards usually do not experience this problem” (P3).*
Personal constraints	➢ Low knowledge level. ➢ Perception of contracting cancer. ➢ Low Job motivations.	*“I remembered my first experience with a child who was diagnosed with bleeding Retinoblastoma. I had to battle with lots of thoughts such as: Do I have to put pressure on the eye a little bit? So how often was I going to be changing the dressing and how am I going to be giving the morphine? how to even maintain the dressing was a problem.” (P1)*

Authors Construct (2020)

### Administrative constraints

Administrative constraints in this study refer to challenges in caring for children with cancer that results when the organization did not provide adequate structural and functional logistics in the hospital environment. Majority of the nurses experienced administrative challenges such as Time-consuming care, lack of teamwork, inadequate logistics, work stress and reduced labour force. The challenges contributed to the feeling of job dissatisfaction among nurses.

#### Time-consuming care

The participants mentioned how intense the period used in taking for children with cancer is. To care for children with cancer. Participants had to sometimes work overtime.

A participant narrative concerning time-consuming care is as follow:

*“How do I manage my time? The problem is often not me; it relates to the manner of the oncology nursing care too. Many of us had experienced staying at the hospital even after our shift ended. I am often compelled to come to the hospital during my day-off. I feel it may be related to the time-demanding nature of oncology care. I feel that my colleagues in other hospital wards usually do not experience this problem” (P3).*

#### Lack of team-work

From the nurses’ point of view, teamwork is a key issue for them in providing care and in some cases, it is not evident in their performance. They noted that having effective teamwork in caring for children with cancer could partly mirror their professional performance. From the participant’s view, most of the oncology nurses often do not have the zeal to go and administer the chemotherapy mediations to the children when the children are due to take their routine chemotherapy medications.

A participant gave narratives about the lack of teamwork by saying:

*“I sometimes do not have interest in the oncology patient. So, when it happens that somebody is in the oncology cubicle and it is time for serving medication, I sometimes totally opt-out. Because of the lack of the zeal to work with my colleagues” (P10).*

#### Inadequate logistics

The availability of modern and adequate equipment and a separate structure dedicated to pediatric cases is one of the essentials of providing useful pediatric oncology care. The lack of equipment can lead to work disruptions, delays and lack of care.

Some participants gave narratives about inadequate logistics by saying:

*“From my opinion, I feel that we don’t have a well-structured unit for oncology patients.” (P1)*

*“I feel that we do not have the equipment to work with the patient. Like face masks, gown, apron, wellington boot. Generally, we lack supply of safety gears and protective clothing” (P3)*

*“Despite the importance of safety in the oncology ward, it seems that there are some limitations in the aspect of providing protective equipment or even the whole ward structure. On countless occasions, I do prepare chemotherapy medications at that time that the drug mixing chamber does not work, or with the absence of a face mask or gown. I can’t stop caring (silence). It’s an issue.” (P5)*

#### Work stress

Job satisfaction is considered a measurement of workers’ contentedness with their psychological, physiological work environmental. The lack of equipment can lead to lack of care and emotional exhaustion for most nurses, as they had to struggle to thoroughly assess the oncology children condition, give chemotherapies and other routine therapies to the children with cancer and at the same time, listen and take care of the demand of the child’s family members. After that, they still had to do other administrative work of documenting all care process carried out on the child.

Some participants gave narratives about *feeling stressed* by saying:

*“I will say it’s very laborious and so involving. Take an example like giving chemotherapy to some of the patients especially at the time that there are many patients in the ward. I could spend about an hour or two on each patient. That means I have to stand the whole day without rest. After which I am required to do documentation and monitor the patient as well. So, it’s so involving and labour-intensive.” (P9)*

*“It is tough, it is tough, I don’t even know what to say, I have never pushed a truck. You have seen those truck pushers, pulling and pushing the truck, I can say caring for oncology case is like that. It is difficult.” (P11)*

#### Reduced labour force

Having sufficient human resources who are available to run shifts is very important because it significantly affects nurse’s morale. Most of the pediatric oncology nurses complained that they are not adequately staffed, this causes loads of work on the few staff in the ward that could contribute to their having low caring morale.

Some participant spoke about the reduced labour force by saying:

*“We don’t have adequate staff.” (P8)*

*“I feel that most people do not willingly want to become Oncology staff.” (P10)*

*“We the nurses are not many, Sometimes I could be away and I will get a phone call from the ward manager. I was ever called that a child is going for chemotherapy, and I had to return to work because of the reduced staff strength” (P11)*

### Personal constraints

Personal constraints of the participants refer to the pediatric oncology care challenges that can be mitigated by to some extent by the nurses themselves. The personal constraint of the clients in this study include their low level of knowledge, perceptions of contracting cancer and Low Job motivations.

#### Low level of knowledge

Having sufficient theoretical, clinical experience and attitude can be significant in improving the morale of the Nurses. Some participants gave narratives about low levels of knowledge by saying:

*“I remembered my first experience with a child who was diagnosed with bleeding Retinoblastoma. I had to battle with lots of thoughts such as: Do I have to put pressure on the eye a little bit? So how often was I going to be changing the dressing and how am I going to be giving the morphine? how to even maintain the dressing was a problem.” (P1)*

*“I feel that to be an oncology nurse, one needs to be a fast learner and be ready to update oneself about new trends with regards to cancer care. Unfortunately, most of us the oncology nurses have many excuses for not been abreast with current happenings about oncology care. I believe that the consequence is that we do not all have adequate training or knowledge on the cancer cases.” (P8)*

*“So, what we do is, we have our number of nurses who have few numbers of workshops on cancer, that with the help of the current pediatric assistant head of department we can manage some of the cases, however, this knowledge is not enough.” (P10)*

#### Perception of contracting cancer

Some nurses in this study also think that they could get cancer as a result of caring in an environment that is not so friendly about putting strategies in place to protect them from being exposed. Some participants gave narratives about their Perception of contracting cancer by saying:

*“A patient on chemotherapy was vomiting and I had to run back to see what was happening to that patient. Yet, while caring for the patient, I was also thinking that what if the drugs splashed into my eyes? what is going to happen to me? What if the drugs get in touch with my skin, what will be the side effects and all that?" (P4)*

*“So, the challenges are so numerous when it comes to even your colleagues, sometimes assigning colleague nurses, to nurse some oncology cases is interesting. You will hear somebody telling you that as for this case I’m scared to go near the person. So, you’ll now ask yourself, if you are scared who should go? So that has been an issue.”* [5]

*“I had medication entering my eyes, I was sad thinking about what the outcome will be in the future, but then I am still moving on, it’s a challenge” (P7)*

#### Low job motivation

Motivation is a concept used to describe the external state that stimulates a particular behaviour and reveal the internal response of that behaviour is interpreted as a stimulus to work, which guides the efforts of workers to achieve organizational goals. The motivation of workers in this study is the result of the interaction between individuals (internal psychological process), their working environment (transaction process) and the fit between these interactions and the social environment. Most participants mentioned that they experienced low job motivation.

A participant also gave narratives about the low Job motivations by saying:

*“Working conditions in an oncological ward needs incorporating supportive environment, but importantly self-motivation. However, in this facility, we do not get such extrinsic motivation. I don’t want to even talk about it because it is often not there. I only get Job satisfaction through my intrinsic self-motivating ability without any external source” (P4).*

## Discussion

The present study aimed to explore and understand the challenges that the pediatric oncology nurses experience whiles caring for children with cancers in Ghana. Two main categories were drawn from the data analysis. The first discussed the Administrative-constraints. These included: Time-consuming care, lack of teamwork, inadequate logistics, work stress and Reduced labour force. The second is related to personal constraints, which included a low level of knowledge, perceptions of contracting cancer and Low Job motivations. These challenges hindered the provision of curative, supportive/palliative, and end-of-life pediatric cancer care in Ghana.

The oncology ward environment is created so that the pediatric oncology nurses could work with a multidisciplinary team to create a curative, supportive/palliative and end-of-life care to children with cancer and their families. In such complex care environments, pediatric oncology nurses mustn’t become task orientated, because of the challenges they face and lose sight of the holistic and human aspects of pediatric oncology nursing caring practice. Accordingly, a study mentioned that it is necessary to pay attention to the barriers that affect oncology care. The study mentioned that by doing so, it leads to improved pediatric oncology patients and families care, as well as, the enhancement of the emotional, social, and educational needs of the nurses [24]. Other studies also opined that all these challenges should be removed regardless of cultural, political, and social differences [25, 26].

In this current study, nurses stated that the hospital managers asked them to be constantly available by the patient bedside to provide care services, but considering the amount of work that must be done in each shift, they often feel overworked and tired. This is because most nurses had to run extra unpaid duty due to the time-demanding nature of the work. They also lamented on low job motivation, saying they don’t receive support from management. They expected that some extra allowances should be provided, but they don’t get that.

The results of this study also showed that there was no well-structured unit dedicated to only the oncology patients. In addition to this, participants complained about inadequate equipment such as face masks and other personal protective equipment. They also reported being stressed, saying that the pediatric oncology nursing work was very laborious, for example giving chemotherapy to some patients especially at the time when the ward is flooded with patients. They mentioned that such occasion demanded that nurses spend about an hour or two on each patient. Which meant, they had to stand the whole day. After this, they still had to do documentation and monitoring as well. To describe this stress, one of the participants linked it with the work of a truck pusher, because of how labour-intensive it was. Majority of the participants also complained about reduced labour force by saying that they were often called back to the ward when they were off duty to come and administer chemotherapy. In line with this finding, a study also found out that there is a shortage of pediatric oncology nurses. As if that is not enough, there is also a shortage of assistant ward aids (trained nurses who does nonspecialized work such as transportation of patients) [27]. Other studies have also confirmed, inappropriate work environments such as nurse shortages, workload, high nurse-patient ratio, overcrowded hospitals, burnout of nurses, and poorly constructed pediatric oncology hospital wards [28–30]. Implementing a minimum nurse-to-patient ratio, will improve patient outcomes, reduce hospital stays, reduce admission rates and reduce the burnout rate of nurses. This is because the shortage of nursing staff in this current study resulted in overworked nurses who were not able to effectively carry out the humanized pediatric care of communicating with patients and paying attention to patients and families care needs.

Participants also acknowledged experiencing some personal constraints such as; low level of knowledge on how to carry out some procedures including wound dressing for children with cancer of the eye (Retinoblastoma) who had constant discharge from the eye. The nurses had difficulty in controlling the pain of such children. Nurses struggled with how frequent they needed to administer analgesics (Morphine) to such children. This was because of the fear of side effects of the medications. These challenges, often create emotional and psychological trauma to the nurses themselves. In line with this current finding, another study also reported that in most schools that train nurses, very little information about oncology is provided. Concurrent with this finding, a recent study showed that the number of nursing schools with oncology majors has been greatly reduced [31].

A study also detailed that lack of knowledge, limited beliefs in cancer care, poor motivation, job dissatisfaction and the inability to give attention to all patients, were the personal challenges experienced by nurses [32]. Other studies also stated, the lack of knowledge to explain all information to patients’ families was a major challenge of nurses [5, 30, 32]. However, contrary to these findings, other studies indicated that nurses around the globe are knowledgeable and play a vital and central role in the delivery of all cancer treatment modalities, principally surgical, radiation, and medical oncology. For patients undergoing surgical intervention, nurses teach patients what to expect before, during, and after procedures [33, 34].

In the current study, pediatric oncology nurses also experienced a lack of teamwork. This finding is in line with a study that acknowledged that there is a lack of interprofessional collaboration and there is a lack of clarity and accurate communication among oncology team members [25, 28]. Participants also mentioned that they believe that their work makes them exposed to getting cancer. A participant narrated an occasion when a patient’s vomitus splashed on her eyes. This ultimately results in anxiety and job dissatisfaction. Concurrent with this finding, previous studies reported, a stressful work environment, increased rate of a medication error and decreased quality of care provided to patients [35, 36]. Some studies have highlighted that in Egypt, nurses and pharmacists were exposed to hazardous drugs for cancer treatment, and also in Iran, nurses showed changes in their mitochondrial parameters and there was cytotoxicity of their lymphocytes due to exposure to chemotherapy inhalation [5, 13]. These unfortunate incidents occurred because the hospital where the participants worked did not have adequate personal protective equipment’s for the nurses to work with. In line with this finding, another study also acknowledged that it is a widely accepted culture to discuss any concerns related to patients’ and staff’s safety [37]. A shared understanding of challenges and appropriate communication of safety concerns among staff in oncology is the key to appropriate oncology care.

### Strengths of the study

The qualitative approach of this study aided us to arrive at an in-depth understanding of the Pediatric Oncology Nurses’ Care Practice Challenges in Ghana as a situation of “Administrative constraints” and “Personal-related constraints”. Thus, it points to the fact that the associated challenges need to be resolved to provide adequate care for the children with cancer and their families and help nurses to gain satisfaction from their work. Additionally, this study is the first qualitative study of this kind, that examined the challenges that the pediatric oncology nurses experience when caring for children with cancer in Ghana. The systematic content analytical steps of Elo and Kyngas proved to be a helpful approach to analyze the research findings because it led to the systematic analysis of the data collected.

### Limitations of the study

This current research did not limit its focus to a precise type or stage of cancer. Thus, the participants worked in oncology wards that had a broad range of children with different types and stages of cancer. Furthermore, the meetings between the research team members during the process of analysis were all conducted using virtual means, hence some critical interpretations about the research findings might have not been adequately discussed. Thus, some levels of differences in the interpretations process might remain among the research team members. However, the Rigor that was maintained throughout the study helped to reduce the effects of the bias that might have occurred.

## Conclusions

Two main categories were drawn from the data analysis. The first discussed the Administrative Constraints that the pediatric oncology nurses encountered in the work environment. The second is related to Personal constraints. These challenges hindered the provision of curative, supportive/palliative, and end-of-life pediatric cancer care in Ghana. Addressing these challenges may require developing strategies that simultaneously address the challenges at the health system, interdisciplinary and individual levels. Such strategies may include strengthening health education and investing in human, material and financial resources for delivering childhood cancer services. Thus, reducing these challenges identified in the current study could result in improved survival and quality of life (QOL) for children with cancer and leads to the nurse’s job satisfaction.

### Recommendations

This study can be retained within the developing body of nursing knowledge on how nurses experience challenges of caring for children with cancer.

Nurse educators should increase the awareness of the challenges entailed in caring for children with Cancer and help suggest ways to mitigate it.

Hospitals should embrace and resolve the needs of nurses working in cancer care unit to better the path of pediatric oncology nursing care in Ghana.

There is the perceived need to enforce strong advocacy policies at both organization and national level to secure funding to improve the hospital working condition to equip it with care support logistics.

Future studies can explore interventions to help overcome the challenges that are impeding nurses from providing high-quality pediatric oncology nursing care in the hospital setting.

## Supplementary Information

**Additional file 1.** The Interview Question Guide. The interview guide file presents a list of questions developed by the research team according to the literature review and considerations of the current study aim. This guide consists of two categories which include the main and the probing qualitative interview questions.

## Data Availability

The transcripts from the interviews data set will be made available upon reasonable request from the corresponding author.

## Abbreviations

COREQ: Consolidated criteria for reporting qualitative studies
TTH: Tamale Teaching Hospital
