# Supplementary material for: A qualitative study of Ghanaian pediatric oncology nurses’ care practice challenges
Source: BMC Nurs. 2021 Jan 12;20:17. doi: 10.1186/s12912-021-00538-x (PMC7802173; doi:10.1186/s12912-021-00538-x)
Supplement: Supplementary file 1 — Additional file 1. The Interview Question Guide. The interview guide file presents a list of questions developed by the research team according to the literature review and considerations of the current study aim. This guide consists of two categories which include the main and the probing qualitative interview questions. [file 12912_2021_538_MOESM1_ESM.docx]

**Additional file 1:**

**Interview Question Guide**

1. Can you please tell me about your thoughts and feelings regarding your work of caring for children with cancer?
2. Tell me about your feelings about the challenges you experience when taking care of children with cancer?”
3. If you want to compare the challenges you experience when taking care of children with cancer with something else, what it is more like?
4. What is/are the meaning(s) of these challenges.

**Probing questions**

1. Could you please describe it more?”
2. Can you please give an example?
3. How do these challenges affect you?

**Socio-Demographic Data**

1.Age: …………………………………………….

2.Sex: ……………………………………….

3.Level of Education: ……………………………………….

4. Numbers of years of practising as oncology nurse: ………………………………………
